# “Valha-nos a Providência!”: festas e ritos católicos em tempos epidêmicos, Salvador-BA, Brasil, 1918-1919^*^

**DOI:** 10.1590/S0104-59702024000100009

**Published:** 2024-04-15

**Authors:** Edilece Souza Couto

**Affiliations:** i Professora, Universidade Federal da Bahia. Salvador – BA – Brasil edilece@ufba.br

**Keywords:** Spanish flu, Smallpox, Catholicism, Lay associations, Salvador (Bahia, Gripe espanhola, Varíola, Catolicismo, Associações leigas, Salvador (Bahia

## Abstract

O artigo analisa as reações dos católicos vinculados às associações leigas na cidade do Salvador, no período da gripe espanhola (1918) e da varíola (1919). Os jornais foram as principais fontes utilizadas para a identificação das festas e dos ritos, tanto dos praticados para pedir a intercessão dos santos quanto daqueles que foram suspensos em função da necessidade de isolamento social. Apesar de ambas as doenças serem transmissíveis e do curto espaço de tempo entre as duas epidemias, a análise das fontes evidenciou diferentes reações dos fiéis quanto às medidas de proteção e busca da cura.

## Abstract (en)

This article analyzes the reactions of Catholics linked to lay associations in the city of Salvador, in the period of the Spanish flu (1918) and smallpox (1919). Newspapers were the main sources used to identify the festivals and rites, both those practiced to ask for the intercession of the saints, and those that were suspended due to the need for social isolation. In spite of both diseases being transmissible and the short interval between the two epidemics, the analysis of the sources showed different reactions from the faithful regarding the measures of protection and the search for a cure.

Salvador, 3 de abril de 2020. A imagem peregrina do Senhor do Bonfim saiu em procissão, sem acompanhamento, por ruas e avenidas. Era a primeira procissão em tempo de pandemia. Para evitar aglomeração, apenas um carro levava a imagem sacra para um cortejo solitário. Os fiéis o acompanharam pelas janelas de casas e edifícios. Estenderam toalhas brancas nas janelas e expuseram as imagens dos seus santos de devoção. Os demais viram o préstito pela TV, pelas redes sociais e plataformas digitais. Dessa forma, recebiam as bênçãos e rezavam pelos doentes de covid-19.

A realização daquela procissão suscita algumas questões: por que, dentre tantos santos de devoção dos soteropolitanos, a imagem do Cristo crucificado foi escolhida? Em quais outros momentos de epidemia houve procissões de penitência? Como os católicos reunidos em associações leigas reagiram aos surtos epidêmicos? Este artigo tem por objetivo responder a essas questões. Nos limites de um artigo, não é possível comparar a covid-19 com outras doenças transmissíveis, pois isso demandaria uma pesquisa aprofundada nos documentos já produzidos sobre a pandemia. O relato inicial serve apenas como fio condutor para analisar as práticas religiosas durante a gripe espanhola (1918) e a varíola (1919).

Christiane Souza (2005), ao analisar a gripe espanhola em Salvador, por meio das publicações nos periódicos locais, argumenta que os discursos nessas fontes podem revelar diferentes aspectos da sociedade, como conhecimento médico, políticas de saúde pública, o sistema econômico, “as percepções e representações das doenças e as respostas das pessoas comuns” (p.72). Ao estudar as epidemias em Portugal, de 1854 a 1918, Maria Antónia Almeida (2014) também utilizou os jornais para verificar de que forma o conhecimento científico, principalmente sobre saúde e higiene, chegava aos cidadãos comuns, que, apesar do analfabetismo, tinham acesso às informações não exclusivamente pela escrita, mas também pela oralidade, nas conversas com letrados em tavernas e mercados. Os jornais traziam descrições e representações da vida cotidiana. Da mesma forma, neste artigo, a intenção foi analisar, por intermédio das notícias e dos discursos veiculados nos periódicos soteropolitanos, as reações dos católicos, sobretudo os reunidos em associações leigas (irmandades, confrarias, devoções e ordens terceiras), diante das epidemias de gripe espanhola e varíola e as vivências em festas religiosas em Salvador.

Os jornais *Diário de Notícias, O Imparcial* e *A Tarde* foram as fontes privilegiadas na pesquisa. Os anúncios e convites para as festas religiosas, as narrativas sobre elas e os silêncios (ausência de informações) nos dão um panorama dos festejos realizados ou não em Salvador durante os períodos críticos das duas epidemias. Esses jornais surgiram em momentos distintos. O *Diário de Notícias* foi fundado pelo jornalista Manoel Lopes Cardoso e circulou na capital de 1875 a 1979. Entre 1903 e 1918 pertenceu ao coronel Vicente Lins Ferreira do Amaral, e em 1919 foi vendido ao almirante Requião, professor e deputado federal pelo Partido Social Democrático. O jornal *A Tarde* foi fundado pelo advogado e jornalista Ernesto Simões Filho, em 1912, ano de início do primeiro mandato do governador José Joaquim Seabra e do processo de modernização de Salvador. O fundador esteve à frente do jornal até 1957, ano da sua morte, e o definia como um jornal conservador, fiel à tradição e avesso às mudanças bruscas. Já *O Imparcial* foi fundado em 1918, por Lemos Brito, com a intenção de promover a candidatura de Rui Barbosa à Presidência da República (Santos, 1985).

Segundo Luca (1996, p.95), a partir do final do século XIX, os jornais passaram a utilizar novos e mais eficientes equipamentos e métodos, trazendo inovação na produção e também nas seções dedicadas ao público feminino, aos esportes, ao lazer, aos assuntos religiosos etc. Na Bahia, a imprensa como empreendimento industrial estava representada pelos jornais *Diário da Bahia* (em 1918 pertencia aos herdeiros do ex-governador Severino Vieira), *Diário de Notícias, A Tarde* e *O Imparcial*. Os dois primeiros estavam em atuação desde o final do século XIX e se adaptaram ao modelo de empresa jornalística da fase industrial. Impulsionados pelos ideais de modernidade e civilização, faziam a cobertura do cotidiano de Salvador, além de publicar informações das agências de notícias internacionais. Os diretores procuravam identificar seus jornais com imparcialidade e isenção política. No entanto, é possível identificar os vínculos com as principais lideranças políticas do estado. O governador José Joaquim Seabra fundou o Partido Republicano Democrata da Bahia, e o jornal *O Democrata*, porta-voz do partido (Sarmento, 2011). O três jornais, cujas matérias utilizamos como fontes, eram dirigidos por adversários de Seabra.

Para averiguar a aplicação das medidas sanitárias e profiláticas, o número de hospitalizações e óbitos em tempos epidêmicos, também recorremos aos relatórios dos provedores da Santa Casa de Misericórdia da Bahia, que, em função da prática de caridade da Irmandade, atendia a população pobre e doente no Hospital Santa Izabel. Não basta contabilizar o número de óbitos, é preciso também analisar as práticas e vivências sociais para se apreender o impacto das doenças em determinado período histórico.

O artigo está dividido em três seções. Na primeira são apresentadas as principais reformas urbanas realizadas em Salvador visando modernizar e sanear o espaço público e civilizar os costumes. Em seguida, são discutidos os limites da modernização com a identificação dos principais problemas de saúde pública, sobretudo os surtos epidêmicos. Há uma vasta bibliografia sobre epidemias, suas causas, formas de tratamento, discursos médicos, práticas de cura, respostas oficiais e as consequências demográficas, políticas e socioeconômicas em diferentes contextos históricos, na Bahia (Souza, 2005, 2009; Souza, Hochman, 2012), em outros estados brasileiros, especialmente no Rio de Janeiro e em São Paulo (Goulart, 2005; Bertolli Filho, 2003; Bertucci-Martins, 2005), e no mundo (Almeida, 2014; Cartwrigh, Biddiss, 2005; Delumeau, 1993; Phillips, Killingray, 2003). Embora as publicações citadas façam importantes análises dos fatores políticos, econômicos e sociais em tempos epidêmicos, abordam de forma mais superficial o impacto dessas doenças nas crenças e o papel das instituições religiosas na organização do culto e na perpetuação de valores como a salvação das almas. Assim, a terceira seção analisa as reações às epidemias por parte de um grupo específico, os católicos reunidos em associações leigas.

## “Em torno dos melhoramentos”: a cidade modernizada

Na Primeira República, Salvador passou por inúmeras intervenções urbanas com o intuito de transformá-la em uma cidade moderna e civilizada, nos moldes do Rio de Janeiro e São Paulo. Havia no Brasil a concepção de que a República representava a modernidade e o progresso, e a Monarquia, o atraso e a barbárie colonial e escravocrata. Era preciso usufruir dos avanços científicos e tecnológicos do Oitocentos – como a eletricidade – que permitiram aperfeiçoar a iluminação urbana, fazer circular os bondes elétricos, melhorar a comunicação por meio de rádio, telégrafo e telefone (Costa, Schwarcz, 2000). Para isso, era imprescindível abrir novas vias e instalar fios e cabos elétricos. Aderir à modernidade e à civilização significava também sanear a cidade, afinal, persistiam os problemas que afetavam a saúde pública, como a má qualidade da água, a deficiente coleta de lixo, as precárias condições de higiene, as aglomerações em antigos casarões e cortiços. O resultado era a alta taxa de mortalidade agravada pelas constantes erupções de doenças transmissíveis.

Desde a gestão do governador Severino Vieira (1901-1904), importantes medidas foram tomadas para aperfeiçoar o serviço sanitário. Foi estabelecido o Regulamento do Serviço Sanitário, que viabilizava a criação da Inspetoria Geral de Higiene, o Instituto Bacteriológico, o Instituto Vacinogênico, o Serviço Geral de Desinfecção e o Hospital de Isolamento. No arrabalde de São Lázaro, na Cidade Alta, foi criado um isolamento para doentes de varíola e uma enfermaria para os acometidos de febre amarela. E em Monte Serrat continuavam os serviços de desinfecção e posto de observação marítimo. Porém, os institutos só tiveram sede própria em 1912, ano em que ocorreu a ampliação do hospital (Souza, 2009).

A primeira gestão do governador José Joaquim Seabra (1912-1916) foi marcada por um projeto de modernização. As obras começaram pelo porto e pela área comercial. Uma nova avenida deveria percorrer toda a cidade, com início no distrito da Sé, passando por Vitória, Barra e Rio Vermelho. Além de facilitar o trânsito dos bondes elétricos, a avenida 7 de Setembro seria o cartão-postal de Salvador (Couto, 2017). Apesar de muitas autoridades civis e religiosas e profissionais da imprensa apoiarem essas intervenções, muitas eram as críticas à modernização, que também modificaria hábitos e costumes que caracterizavam a antiga capital colonial. Encontramos nos jornais muitas narrativas dos problemas enfrentados pelos moradores de Salvador. A matéria “Em torno dos melhoramentos”, do *Diário de Notícias*, por exemplo, elenca alguns desses problemas:

Somos os tristes moradores de uma capital onde existem todos os inconvenientes da vida em sociedade, sem nenhuma das suas vantagens; vida cara, desde os aluguéis das habitações, até as exigências do vestuário; população densa; casas aglomeradas, mal arejadas e ... pelo outro lado, quanto a vantagens, nada, desoladoramente, nada.

Cerca de 300.000 pessoas quase morrendo de tédio, sem diversões nem logradouros públicos e, ainda a sombrear-lhes o espírito atribulado, a preocupação dos males epidêmicos que vão ceifando, assustadoramente, a vida, tal como a febre amarela, o mal levantivo, a varíola, a disenteria e, com uma fúria inominável, a tuberculose que leva para o seu ativo parte dos serviços do nosso obituário (Em torno..., 25 jun. 1912, p.1).

O tempo passava, a execução das obras atrasava e os soteropolitanos conviviam com os entulhos das demolições, ruas e avenidas esburacadas e sujas, o que causava transtornos na mobilidade e a propagação de doenças. Assim, em Salvador, o novo e o velho interagiam. As construções e as manifestações culturais e religiosas da metrópole colonial coexistiam com novas avenidas, os bondes elétricos e os fogos de artifício, símbolos do progresso.

As associações leigas católicas também procuravam adaptar-se à modernização. A Irmandade da Santa Casa de Misericórdia da Bahia, responsável pelos serviços de assistência aos órfãos, presos e doentes entre a população considerada indigente, procurou realizar reformas nos seus prédios para melhorar as condições sanitárias. A rua da Misericórdia foi alargada em 1915. E a irmandade construiu um posto de observação para o isolamento de pessoas com suspeita de doença contagiosa (Santos, 1918, p.89). Havia um cuidado especial com os mortos. Desde 1912, a Misericórdia tinha um contrato com a Companhia Linha Circular de Carris da Bahia para o transporte de cadáveres de indigentes do Hospital Santa Izabel para o Cemitério do Campo Santo. Esse serviço também necessitava de melhorias, pois muitas vezes os corpos chegavam ao cemitério sem certidão de óbito e só eram inumados quando já estavam em franca decomposição (Santos, 1918, p.57-89).

Quando o articulista do *Diário de Notícias* afirmava que os soteropolitanos viviam “quase morrendo de tédio, sem diversões”, revelava que ainda eram escassas as possibilidades de entretenimento na cidade. A população encontrava no Teatro São João e em algumas salas de cinema as poucas exposições, os raros concertos, espetáculos e exibições de películas que promovessem os encontros e divertimentos. A maior parte da sociabilidade ainda era vivenciada nas igrejas e nos largos nos dias de festas dos padroeiros, em banquetes, leilões e jogos promovidos por irmandades, confrarias e ordens terceiras.

## “A cidade hospital”: as epidemias e os limites da modernização

Apesar das intervenções urbanas em Salvador, os problemas de saneamento e surtos epidêmicos persistiam. O jornal *A Tarde*, ao relatar os casos de varíola em 1919, chamou a capital de “cidade hospital” (A cidade..., 1 nov. 1919, p.1). Houve decréscimo populacional. Em 1912, a cidade contava com 348.130 habitantes. Esse número caiu para 283.422 no registro do censo de 1920 (Recenseamento..., 1926). É claro que várias foram as *causa mortis* dos 64.708 soteropolitanos, mas é preciso considerar o alto índice de hospitalizações e mortalidade por doenças transmissíveis nesse período.

Em novembro de 1917, o *Diário de Notícias* (Os óbitos..., 9 nov. 1917, p.2) publicou o “Boletim Demográfico Sanitário” do mês anterior, divulgado pela Secretaria Geral de Saúde Pública. De acordo com o boletim, ocorreram 96 mortes causadas pelas seguintes doenças: tuberculose pulmonar, 18 óbitos; diarreia e enterite, 14; mal de Bright (insuficiência renal crônica), uremia e arteriosclerose, seis óbitos cada; afecção orgânica do coração, quatro; senilidade, debilidade congênita, cirrose do fígado e impaludismo agudo, três óbitos cada; tétano, hemorragia cerebral, apoplexia, convulsões infantis, bronquite aguda e infecção intestinal, dois óbitos cada; varíola, sarampo, tuberculose, meningite, meníngea, sífilis, câncer de língua, aneurisma, broncopneumonia, pneumonia, apendicite, nefrite, gangrena e onfalite infecciosa, um óbito cada. Também foram registrados três óbitos por suicídio com veneno; um por submersão acidental (afogamento) e um homicídio por instrumento contundente.

A segunda doença a causar maior número de mortes foi diarreia. Dos 14 óbitos por diarreia e enterite, 11 eram de crianças com idade abaixo de 2 anos. Problemas intestinais podem causar diarreia ou uma inflamação nos intestinos (enterite) e podem ocorrer em função do consumo de alimentos e água contaminados por bactérias e vírus. Essas duas doenças estão relacionadas à precariedade das condições sanitárias nas áreas urbanas. Dessa forma, o ideal de cidade moderna e civilizada encontrava os seus limites.

Além do número notificado de óbitos por doenças transmissíveis (tuberculose: 18; impaludismo: três; e varíola: um), a mesma matéria ainda informava o número de pessoas internadas (14, sendo nove com beribéri, três com varíola e dois em observação) no Hospital de Isolamento. Nos dois anos seguintes, nessa listagem seriam acrescentadas duas epidemias: *influenza* (1918) e varíola (1919). A última parecia controlada em outubro de 1917, com apenas um caso confirmado e três suspeitos.

Cristiane Souza (2009, p.53) elaborou um quadro referente à mortalidade por moléstias transmissíveis em Salvador, de 1908 a 1919. Foram identificadas mortes por tuberculose, impaludismo, varíola, gripe, disenteria, febre amarela, tifo, peste, sarampo, difteria, beribéri, lepra, coqueluche e escarlatina. Dessas doenças, a tuberculose e o impaludismo tinham, a cada ano, o número de vítimas acima de três centenas. Por tuberculose, o número de óbitos teve variação de 756, em 1908, a 1.065, em 1919; o impaludismo, de 319 a 532 óbitos. A gripe matou de 8 a 28 pessoas anualmente antes de 1918, ano em que o número subiu para 386, devido à gripe espanhola. No ano seguinte, caiu para 48, sendo ainda muito superior aos anos anteriores. A varíola, que teve surtos em 1909 (328 óbitos) e 1910 (835 óbitos), teve 2.804 óbitos em 1919.

Sabemos que o número de mortes é aproximado, em função dos diferentes métodos aplicados por diversas instituições de registro. Por isso, pesquisamos também os registros nosográficos do Hospital Santa Izabel, anexados aos relatórios dos provedores da Santa Casa de Misericórdia da Bahia para a identificação das principais moléstias apresentadas pelas pessoas hospitalizadas anualmente, de 1914 (dois anos após as primeiras intervenções urbanas) a 1919 (ano da epidemia de varíola).

**Figura 1:**
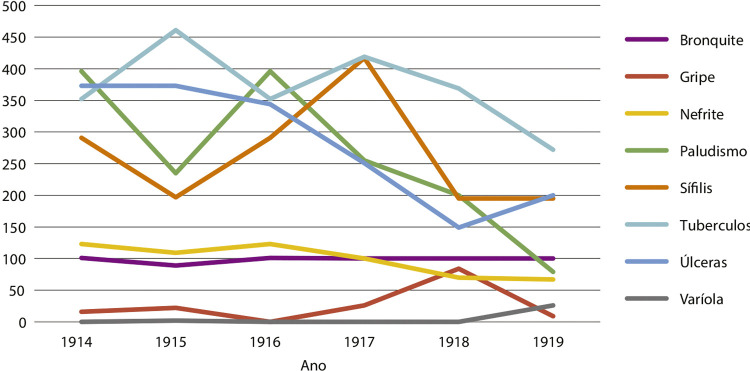
: Hospitalizações por doenças transmissíveis, 1914-1919 (Registros nosográficos da diretoria do Hospital Santa Izabel nos relatórios dos provedores da Santa Casa de Misericórdia da Bahia, 1914-1919)

O gráfico demonstra que a tuberculose e o impaludismo foram as principais causas das internações hospitalares. No período analisado, de 272 a 461 tuberculosos estiveram em tratamento; de 79 a 396 internados sofriam de impaludismo; de 16 a 26 pessoas foram internadas com gripe, sendo que esse número chegou a 84, em 1918, e caiu para nove, em 1919. O número de doentes de varíola foi pequeno, de zero a dois, até 1918, e chegou a 26 no ano da epidemia. Chama a atenção, nesse período, a incidência de doentes de sífilis, com variação de 195 a 417 casos, e bronquite com média de uma centena de casos anuais.

A tuberculose pulmonar, maior causa de morte e de internações hospitalares entre os soteropolitanos no período de 1908 a 1919, era uma preocupação constante. As habitações necessitavam de limpeza e ar puro. Porém, a clareza dos males causados pelo acúmulo de sujeira e entulhos nas ruas e pela aglomeração em espaços pouco ventilados demorou a se manifestar nas mentes das autoridades públicas, mesmo com o alerta de que havia um surto de gripe em Salvador em setembro de 1918.

A situação deficitária da saúde pública na primeira metade do século XX, entretanto, não era uma exclusividade baiana. No Rio de Janeiro também eram precárias as condições de higiene e habitação. E não houve preocupação com os casos de morte por gripe até setembro de 1918, quando morreram 48 pessoas. Acreditava-se que fosse mais um surto de gripe que matava idosos. No entanto, a maior parte dos óbitos acontecia entre pessoas de 20 a 40 anos. Em outubro o número de óbitos foi 930. Na capital federal, estima-se que 15 mil pessoas foram vitimadas pela *influenza*. Entre os problemas enfrentados pelos cariocas, os jornais elencavam os de maior gravidade: escassez de verbas direcionadas à saúde pública, precária inspeção no porto, falta de estrutura para atendimento hospitalar e deficiente formação dos enfermeiros (Goulart, 2005).

Em Portugal, cidades, como Porto, eram insalubres. Os operários partilhavam quartos sem água encanada e saneamento básico. A gripe de 1918 encontrou a população debilitada por outras doenças como cólera (1850), peste bubônica (1899) e tifo (1917). A propagação da gripe pelo ar fez com que as autoridades determinassem a higienização das habitações, o isolamento dos doentes, a suspensão das aulas e a interdição de feiras e mercados. Assim como na Bahia, a epidemia teve seu pico em outubro e declínio em novembro. Porém, entre os meses de junho e dezembro de 1918, os portugueses precisaram combater outro surto epidêmico: de varíola. A vacina, não obrigatória pela lei, passou a ser imposta pela necessidade (Almeida, 2014).

Enquanto Portugal perdia cidadãos para a *influenza*, a varíola e a Primeira Guerra Mundial, os brasileiros acompanhavam as notícias europeias sobre a gripe e a guerra como se fossem problemas distantes. Jean Delumeau (1993, p.117-118), ao analisar o medo das pestes na Europa, da Idade Média ao século XIX, afirma que, quando há perigo de contágio, a primeira reação, tanto das autoridades como das pessoas comuns, é não ver, e a inércia se repete, independente de qual seja a doença, com a justificativa de não alarmar a população e não interromper as transações econômicas, não promover a ruína dos negócios e o desemprego. Além da existência de um efeito psicológico inconsciente, o medo da doença faz com que se considere melhor esperar pela regressão do mal por si mesmo.

No Brasil de 1918, enquanto as autoridades preferiam considerar a gripe uma doença benigna e protelar as medidas de proteção, três grandes cidades portuárias, Rio de Janeiro, Salvador e Recife, receberiam o vírus da gripe espanhola por intermédio do paquete inglês Demerara. Segundo Souza (2005, p.82), “as autoridades pareciam preferir negar o fato” e criticavam a imprensa de oposição ao governo por disseminar notícias alarmantes, quando se tratava apenas de uma gripe, sem maiores consequências, que periodicamente atingia a população baiana. Apesar de o número de casos diagnosticados de gripe ter aumentado a partir do mês de setembro, só na primeira quinzena de outubro foi criada uma comissão médica para verificar a situação sanitária. E os médicos concluíram que não era nada grave, nada além de uma doença familiar e benigna, para a qual sugeriam algumas medidas profiláticas, como a desinfecção de lugares com muito trânsito e aglomeração de pessoas e a higiene individual.

As medidas sugeridas não foram executadas, e os jornais continuavam publicando o número de mortos, informações sobre os distritos mais afetados e denunciando a morosidade das autoridades públicas para sanear a cidade e conter a transmissão do vírus. Na falta de medidas mais enérgicas contra o contágio, os articulistas sugeriam o fechamento de teatros, casas de espetáculos e cinemas para evitar aglomerações. Assim conclamava o *Diário de Notícias* (Gripe, 18 out. 1918, p.1). Como resposta ao alarme da imprensa, o diretor de Saúde Pública, Muylaert (23 out. 1918, p.1), pediu a publicação da seguinte carta no mesmo jornal:

Bahia, 21 de outubro de 1918. – Ilmo. sr. redator do Diário de Notícias – A notícia, publicada hoje em vosso conceituado jornal, [de que] ‘pela cidade, a epidemia continua se alastrando pavorosamente’ não está de acordo com os dados colhidos pelos inspetores sanitários por solicitação minha, em colégios, quartéis, e demais casas coletivas e informes das principais farmácias, no que se refere ao receituário, pois verifica-se o decrescimento sensível da gripe. Espero que fareis a necessária retificação, para tranquilidade da população, com que muito obsequiareis o vosso leitor. – A. Muylaert.

Um mês após a confirmação dos casos e das mortes causadas pela gripe espanhola, a Diretoria Geral de Saúde Pública (DGSP) resolveu tomar algumas providências, principalmente a desinfecção dos espaços públicos: ruas, mercados, elevadores, estações e bondes de transporte de passageiros e mortuários. Igrejas, centros espíritas e terreiros de candomblé não foram interditados. E foi instalada uma enfermaria no Hospital de Isolamento de Mont Serrat. O jornal *O Democrata* (Gripe, 10 nov. 1918, p.1) informava que 216 indigentes foram infectados pelo vírus da gripe e atendidos de forma gratuita pela DGSP. Porém, é provável que o número de vítimas entre a população pobre fosse bem maior, pois os jornais traziam muitos relatos de pessoas que morriam nas ruas ou nas proximidades dos hospitais, sem atendimento, por falta de vagas.

Ainda não refeitos da epidemia de gripe espanhola, no ano seguinte, 1919, os soteropolitanos tiveram que enfrentar outro vírus, o da varíola, doença conhecida por seus efeitos devastadores desde o período colonial. A vacina era obrigatória no Brasil desde o século XIX, porém, a falta do imunizante e a resistência à vacinação são fatores que devem ser considerados para entender os surtos epidêmicos no século XX. Embora na Primeira República a Bahia tenha tido registros de casos e mortes por varíola todos os anos, segundo Souza e Hochman (2012, p.5), os mais críticos foram 1909, 1910 e 1919, ocorrendo em 1919 a epidemia “mais devastadora que a Bahia conheceu: entre junho e dezembro do referido ano, 4.612 pessoas foram acometidas, e 2.804 foram vitimadas pela doença”. Apesar de febre amarela, malária e tuberculose também fazerem parte do quadro de doenças que ceifavam muitas vidas na capital da Bahia, os surtos seguidos de *influenza* e varíola alarmaram a população, pois não houve tempo para que as famílias se refizessem das perdas econômicas e humanas.

Em 1919, a varíola chegou à cidade do Salvador por intermédio do Exército, quando soldados que estavam em expedição no oeste da Bahia voltaram à capital. Apesar do isolamento no Hospital Militar, no mês seguinte foram identificados 17 casos nos bairros de Brotas e Pilar, localizados, respectivamente, na Cidade Alta e na Cidade Baixa. Ou seja, o vírus circulava pelas regiões mais populosas. Em agosto, os distritos centrais (Sé, Paço, Santo Antônio Além do Carmo, Santana, Taboão – importante ligação entre as partes alta e baixa da cidade) já estavam infectados, assim como os arrabaldes (Souza, Hochman, 2012).

Dessa forma, no segundo semestre de 1919, o medo e o horror estavam estampados nas páginas dos periódicos de Salvador em palavras, representações e imagens. Varíola, variolosos, peste, infestação, violenta epidemia, proporções horríveis e assustadoras são algumas das expressões usadas de forma recorrente pelos articulistas naquele ano. Eram publicadas fotos dos locais de isolamento e das procissões penitenciais. E as representações da morte (desenhos, fotos de caveiras, esqueletos e anjos) eram utilizadas para demonstrar o medo que pairava sobre a cidade. O medo das pestes leva ao enclausuramento e à rejeição dos doentes, mas também se desenvolvem a compaixão e os laços de solidariedade (Duby, 1999, p.78-91).

A varíola era doença com alta taxa de transmissão e letalidade, com cerca de 30% dos óbitos na época. Era transmitida pelas secreções e saliva dos infectados disseminadas por gotículas e aerossóis. Eram formadas pústulas que continham um líquido amarelo semelhante ao pus e onde se alojava o vírus. Os primeiros sintomas eram parecidos com os da gripe: febre, dor de cabeça e no corpo e prostração. Porém, o avanço da doença apresentava náusea, vômito, pústulas e coceira (Cartwright, Biddiss, 2005, p.83-84).

E, assim, “A asa negra da morte paira por sobre toda a cidade”, como alerta o *Diário de Notícias* de 7 de novembro. Nessa matéria, o articulista elenca as providências (abertura de postos de vacinação) e tece críticas ao estado de miséria de grande parte dos moradores da capital, que “morrem ao desamparo, atirados às sarjetas das ruas” e afirma que os cadáveres passavam de 30 a 36 horas nas casas ou nas ruas à espera de remoção, o que contribuía para a contaminação dos vivos e sadios. E ainda cobra providências das autoridades civis para o asseio dos antigos casarões com cômodos repartidos e alugados para diferentes famílias. Essas casas, “pardieiros velhos e sujos”, nas quais habitavam de dez a vinte pessoas, tornavam-se “focos terríveis da varíola”. Quando um varioloso morria, no mesmo cômodo já se encontravam de três a quatro doentes, “uma promiscuidade horrível” (A asa..., 7 nov. 1919, p.1).

Diante do quadro de medo e horror da varíola, do alastramento da doença, da dificuldade de vagas em isolamentos e da falta de controle da epidemia por parte das autoridades sanitárias, a população direcionava seu clamor a Deus e aos intercessores, os santos, principalmente àqueles cujas hagiografias relatavam experiências com as doenças contagiosas.

## “Valha-nos a Providência”: atos penitenciais contra as epidemias

O clamor do jornal *O Imparcial* em 20 de junho de 1919 (Valha-nos... 20 jun. 1919) demonstra uma situação vivenciada em diversos momentos de epidemias. O medo, o desespero e a angústia levam os devotos, e até mesmo as pessoas que alegam falta de fé, em direção ao sagrado. Entretanto, a mesma atitude pode ser observada em escalas diferentes, a depender do grau de gravidade da doença e das informações divulgadas pelas autoridades políticas e de saúde pública e pelas agências de comunicação.

Desde o período colonial brasileiro, os leigos católicos, vinculados às irmandades, confrarias, devoções e ordens terceiras, eram os principais responsáveis pelo culto aos santos, pela promoção das festas e procissões e pelos ritos fúnebres. O clero apenas realizava os sacramentos e celebrava as missas. Além da importância dessas instituições para a expansão da fé, elas se converteram em espaços de sociabilidade, solidariedade, caridade e formação de identidades étnicas (Couto, 2017).

A Primeira República foi um momento difícil para muitas associações leigas, principalmente para as que não tinham igreja própria e ocupavam os altares laterais de outros templos. Algumas importantes igrejas foram destruídas para a execução das obras de engenharia urbana. As igrejas da Ajuda e de São Pedro Velho foram demolidas em 1912 e 1913, respectivamente; a da Sé, em 1933. A nova igreja de São Pedro foi construída na esquina da avenida 7 de Setembro com a praça da Piedade, e a inauguração aconteceu em 1917. E a nova igreja da Ajuda foi construída em um terreno em frente ao antigo templo e inaugurada em 1932. As associações foram realocadas em outras igrejas, como na Sé e na igreja da Palma, o que gerou transtornos e mudanças nos roteiros das procissões e festas (Couto, 2017). Nas crises epidêmicas, muitos dos devotos estavam destituídos dos seus altares, suas alfaias e imagens sacras, o que dificultava as expressões públicas de devoção.

Ao comparar as festas e os ritos (missas, procissões) realizados pelos irmãos de fé em 1918 e 1919, identificamos a recorrência à intercessão divina, mas também diferentes formas de lidar com as duas epidemias. Como já demonstrado, o agravamento das doenças ocorreu entre outubro e novembro. No Quadro 1 elencamos as atividades religiosas realizadas nesse período e seus organizadores. Apesar da ampla divulgação nos jornais da circulação do vírus da *influenza*, as festas religiosas se desenrolavam em igrejas, adros, largos e vias da cidade.

**Quadro 1:** : Atividades religiosas realizadas entre setembro e dezembro de 1918 em Salvador (BA)

Data	Festa/rito	Associação leiga/grupo de fiéis
**Outubro**		
20	Festa de Santa Tereza de Jesus	Venerável Ordem Terceira do Carmo
22	Festa de Nossa Senhora da Piedade	Capuchinhos e fiéis da Igreja da Piedade
**Novembro**		
12	Bando anunciador da Festa de Sant’Ana do Rio Vermelho	Comunidade de pescadores e veranistas do Rio Vermelho
**Dezembro**		
1	Festival preparatório da festa de Reis na Penha	Comunidade e fiéis de Nossa Senhora da Penha, península do Itapagipe
4	Missa festiva para santa Bárbara na Igreja da rua do Paço	Comerciantes do Mercado de Santa Bárbara
8	Missa festiva de Nossa Senhora da Conceição do Boqueirão Festa de Nossa Senhora da Conceição da Praia	Venerável Ordem Terceira de Nossa Senhora do Boqueirão Irmandade do S.S. Sacramento e N.S. da Conceição da Praia
13	Festa de santa Luzia Festa de Nossa Senhora da Conceição dos Pobres	Devoção de santa Luzia na igreja da Saúde comunidade da Caixa d’Água, bairro Cruz do Cosme
22	Missa festiva de Nossa Senhora da Ajuda	Irmandade do Senhor Bom Jesus dos Passos e Vera Cruz
24	Missa de Natal	Venerável Ordem Terceira do Carmo
29 a 31	Eventos preparatórios para a festa de Reis	Comunidade da igreja da Lapinha
31	*Te Deum* e quermesse	Irmandade do Santíssimo Sacramento e Nossa Senhora da Conceição da Praia Venerável Ordem Terceira do Carmo

Fontes: Jornais *Diário de Notícias, O Imparcial* e *A Tarde* (1917-1919); Compromissos das irmandades, confrarias e ordens terceiras – Arquivo da Cúria Metropolitana de Salvador – Laboratório Eugênio Veiga.

Por meio do jornal *Diário de Notícias*, a Venerável Ordem Terceira do Carmo, desde o dia 16 de outubro de 1918, anunciava a festa de santa Tereza de Jesus. No interior do templo, haveria celebração de missas, crisma e *Te Deum*, assim como a inauguração de panteão. E, no palanque montado no adro, haveria apresentações musicais e foguetório. No dia 21, o mesmo jornal elogiava a festa, realizada “com a pompa habitual”, com “numerosos fiéis que enchiam o majestoso templo, caprichosamente ornamentado”. Durante o *Te Deum*, observou-se o espaço repleto, “sendo pequeno o grandioso templo para conter o número de fiéis” (Venerável..., 16 out. 1918, p.3).

Nem bem terminou a festa de santa Tereza, os católicos já se preparavam para homenagear Nossa Senhora da Piedade. O *Diário de Notícias* anunciava os preparativos na igreja dos Capuchinhos para a festa do dia 22, “com grande pompa”: missas das quatro às onze da manhã. O *Te Deum* seria celebrado às 18 horas, e teria a benção do Santíssimo Sacramento. À noite, a banda da polícia tocaria na “iluminada” praça da Piedade, em frente à igreja (N.S. da Piedade, 21 out. 1918, p.1).

Em novembro, chamam atenção as notícias de dois eventos, o cortejo do bando anunciador da festa de Sant’Ana, no Rio Vermelho, e o festival preparatório para a festa de Reis, na Penha. Esses preparativos se convertiam em verdadeiras festas antecipadas para o anúncio da programação das homenagens aos santos, o chamado da comunidade para a distribuição de tarefas e a arrecadação de fundos para as despesas. Ora, em tempo de epidemia, eventos preparatórios para as grandes festas poderiam ser adiados sem prejuízos; afinal, as apresentações dos ternos de Reis ocorreriam da semana do Natal até o dia 6 de janeiro, e a festa de Sant’Ana tinha data móvel entre janeiro e fevereiro. Em 1919, por exemplo, a festa só aconteceu no dia 25 de fevereiro. Portanto, não havia urgência.

Não houve, provavelmente, muita preocupação por parte dos moradores do Rio Vermelho e da Penha com a gripe espanhola, por se tratar de dois arrabaldes, zonas distantes do perímetro urbano, à beira-mar, com fama de bons ares, favoráveis à saúde e com baixa incidência de casos e morte por *influenza*. O distrito da Penha teve o registro de 8% das mortes, enquanto o Rio Vermelho nem aparece na percentagem de mortalidade (Souza, 2005, p.92). Além disso, novembro foi considerado pelas autoridades civis o fim da crise e de afirmação de que, na Bahia, a doença foi mais benigna e menos mortífera do que em outros estados brasileiros. Em 23 de novembro, a Devoção do Senhor do Bonfim, sediada no distrito da Penha, convidou os fiéis para uma missa “em ação de graças pela terminação da ‘Gripe’” (Senhor..., 23 nov. 1918, p.2), realizada às nove horas do dia 24.

Na mesma edição do *Diário de Notícias*, porém, na coluna ao lado do anúncio da missa de ação de graças na igreja do Bonfim, há uma matéria, “A *influenza* no mar”, que revela preocupação com a zona portuária, pois navios com passageiros doentes continuavam aportando em Salvador. O articulista relatava que um paquete inglês identificara um surto de gripe espanhola com 54 casos na terceira classe no quarto dia de viagem, sendo 14 pessoas vitimadas pela doença, e os corpos jogados no mar. Quando chegou a Salvador, 26 dias depois, o navio contava com cinquenta passageiros em primeira classe, 152 em segunda e 336 em terceira. Era grande, portanto, a aglomeração. Desses viajantes, nove estavam doentes e internados na enfermaria de bordo. As autoridades sanitárias, responsáveis pelas vistorias no porto, só permitiram o desembarque de três pessoas que tinham Salvador como destino. Logo abaixo, na mesma coluna, há um anúncio do remédio Triphol afirmando que o uso após a gripe trazia benefícios “remineralizando o organismo, levanta com presteza as forças, tonifica o sistema nervoso abatido, varrendo os restos da doença (A influenza..., 23 nov. 1918, p.2).

Utilizando dados estatísticos oficiais, Souza (2005, p.93) contabilizou os números de infectados e mortos pela gripe espanhola até 30 de novembro de 1918. Dos 320 mil habitantes de Salvador, 130 mil estiveram doentes, e 338 morreram. Assim, 40,6% da população foi contaminada pelo vírus, e a cidade teve uma média de 5,2 óbitos por dia. Com a divulgação do fim da epidemia, muitos católicos, apesar da dor da perda de entes, familiares e amigos, preferiram silenciar o assunto e render graças pelas vidas poupadas. Dessa forma, o mês de dezembro foi pleno de festas.

Por intermédio do *Diário de Notícias* do dia 3 de dezembro de 1918, sabemos que duas importantes festas do mês de dezembro seriam reduzidas a missas. A nota publicada pelos comerciantes do mercado de Santa Bárbara informa apenas que haveria a celebração da missa festiva (Festa..., 3 dez. 1918, p.2). Não há qualquer referência aos festejos com música, dança e banquete de comida afro-brasileira no estabelecimento comercial. É provável que a festa pública tenha sido suspensa. A secretaria da Ordem Terceira de Nossa Senhora da Conceição informava que, “não realizando este ano a festa solene ... porém, mandará celebrar missa às 4 horas da madrugada, 8 e 10, sendo esta a festiva” (N.S. da Conceição..., 3 dez. 1918, p.2), mas não deixa claro o motivo dessa mudança.

Santa Luzia foi festejada em dois espaços, na igreja do Pilar, Cidade Baixa, e na igreja da Saúde, Cidade Alta. De acordo com a programação, no Pilar, missas seriam celebradas a cada hora, das quatro às 11 horas da manhã. A missa solene contaria com um coro de quarenta professores. Também seria celebrado o *Te Deum*, finalizado com queima de fogos de artifício. No coreto, “havendo embandeiramento e iluminação”, a banda da polícia tocaria para animar os devotos (Devoção..., 10 dez. 1918, p.2). O mesmo jornal, em19 de dezembro, informava que “correram esse ano muito animadas as festas de Santa Luzia na Saúde”. Houve “brilhantismo”, sendo que “a concorrência ao local foi desusada” (Devoção..., 19 dez. 1918, p.2).

A edição do *Diário de Notícias* (Festas..., 24 dez. 1918, p.1) da véspera do Natal estampou em sua primeira página as informações sobre as comemorações do nascimento de Jesus que prometiam “grande brilho e animação”. A partir da meia-noite seriam celebradas missas “em todas as igrejas matrizes da cidade”, além das “missas campais em diferentes distritos. Ressaltava que a igreja do Carmo prometia ter a solenidade “das mais brilhantes, para isso não tendo poupado esforços o prior da respectiva irmandade”. Fechava a nota afirmando que, na manhã do dia 25, um clube da cidade realizaria uma “matinê infantil para a distribuição de mimos e para a festa da árvore de Natal, devendo seguir-se animada hora de diversões, em que se farão danças e surpresas”. O final do ano chegava cheio de esperança de dias melhores e com celebrações de ação de graças pela assinatura do armistício da Primeira Guerra Mundial.

O alívio do fim da epidemia de gripe espanhola, entretanto, não durou muito. Seis meses separam esse surto de outro, o de varíola, que assolou a cidade no período de junho a dezembro de 1919. Passado o São João, de julho a setembro não há muitas festas de largo no calendário católico de Salvador. Por isso, escolhemos o período de outubro a dezembro para a análise das celebrações que ocorreram ou foram suspensas durante a epidemia de varíola. O Quadro 2 traz as datas, os eventos religiosos e os responsáveis por sua organização no segundo semestre de 1919.

**Quadro 2:** : Atividades religiosas realizadas entre setembro e dezembro de 1919 em Salvador (BA)

Data	Festa/rito	Associação leiga/grupo de fiéis
**Outubro**		
19	Nossa Senhora do Carmo e santa Tereza	Venerável Ordem Terceira do Carmo
26	Nossa Senhora do Pilar	Irmandade de Nossa Senhora do Pilar
26	Senhor Bom Jesus da Cruz	Confraria do Senhor Bom Jesus da Cruz
**Novembro**		
01	Procissão de penitência para são Roque	Venerável Ordem Terceira de São Francisco
06	Procissão de penitência para o Senhor do Bonfim	Venerável Ordem Terceira de Nossa Senhora do Carmo
14 - 23	Exposição da imagem do Senhor do Bonfim na igreja da Venerável Ordem Terceira de São Domingos	Irmandade do Senhor Bom Jesus dos Passos e Vera Cruz
16	Missa solene para Nossa Senhora da Palma	Irmandade de Nossa Senhora da Palma
20	Missa solene de santa Cecília na igreja da Sé	Irmandade de Santa Cecília
30	Missa de ação de graças pelo livramento da epidemia no arrabalde da Barra	Moradores da Barra e igreja de Santo Antônio da Barra
**Dezembro**		
08	Nossa Senhora da Conceição da Praia Nossa Senhora da Conceição do Boqueirão	Irmandade do Santíssimo Sacramento e Nossa Senhora da Conceição da Praia Venerável Ordem Terceira de Nossa Senhora do Boqueirão
13	Festa de santa Luzia	Devoção de santa Luzia na igreja do Pilar
14	Missa para são Lázaro	Igreja de são Roque e são Lázaro
25	Missa do galo e festa de Natal	Venerável Ordem Terceira de Nossa Senhora do Carmo
28	Missa de inauguração das obras do Cemitério do Carmo	Venerável Ordem Terceira de Nossa Senhora do Carmo

Fontes: Jornais *Diário de Notícias, O Imparcial* e *A Tarde* (1917-1919); Compromissos das irmandades, confrarias e ordens terceiras – Arquivo da Cúria Metropolitana de Salvador – Laboratório Eugênio Veiga.

O *Diário de Notícias* publicou anúncios de três associações leigas que realizaram festas em outubro de 1919. No dia 9, a mesa administrativa da Ordem Terceira do Carmo conclamava os irmãos para que comparecessem à missa e acompanhassem a procissão “com seus hábitos para maior brilho” da festa (Baieiro, 9 out. 1919, p.5). Festejaram também santa Tereza, “com todo esplendor”. Após os festejos, as imagens das santas e a do Senhor do Bonfim continuariam expostas no templo até o dia 26, “a receber as fiéis preces para minorar os sofrimentos da epidemia reinante” (Venerável..., 24 out. 1919, p.3). Ou seja, a aglomeração não foi evitada, mas reforçava-se a necessidade de orações para se obter a intervenção divina contra a varíola.

O *Diário de Notícias*, ainda em outubro, anunciava que no dia 26 haveria missa para Nossa Senhora do Pilar, seguida da procissão, acompanhada pela banda de música da polícia. À noite haveria quermesse, iluminação e música (Religiosas, 25 out. 1919, p.2).

A Confraria do Senhor Bom Jesus da Cruz convidava os fiéis para “a festa do seu orago, como de costume”, no dia 26 de outubro, na igreja da Palma. De acordo com o Compromisso da confraria, fundada por homens pardos^1^ em 1719, cultuava o Cristo crucificado. Reza o artigo 52 do compromisso que a festa do padroeiro deveria ser realizada “no dia 21 de setembro de cada ano; e quando acontecer ser este dia de serviço, se fará a mesma festividade na segunda dominga do mês de outubro, se antes o não poder ser” (Compromisso..., 1914, p.33). Como raramente o dia 21 de setembro caía no domingo, a festa era realizada em algum domingo de outubro. Em 1918, o dia 21 de setembro caiu no sábado; para muitos, um dia de serviço. A festa, então, aconteceu no dia seguinte, domingo. Fato intrigante é que, em 1919, a data era exatamente um domingo; no entanto, os ritos litúrgicos e a festa de largo aconteceram em outro domingo, 26 de outubro, exatamente no momento mais crítico da epidemia de varíola.

A programação da festa do Senhor da Cruz incluía a lavagem da igreja na quinta-feira, dia 23. Ato, por sinal, proibido por portaria de dom Antônio Luís dos Santos desde 1889, “a bem da moralidade, da santidade do culto”. Para evitar a “prática abusiva”, o arcebispo recomendava fazer a limpeza “em dia que não seja quinta-feira, sem anúncio de qualquer espécie que promove ajuntamento, com toda decência e reverência possíveis” (Santos, 9 dez. 1889, p.1). Apesar de ser uma desobediência às regras eclesiásticas, é importante ressaltar que a lavagem de igreja na quinta-feira que antecedia o domingo dedicado ao padroeiro era um momento de aglomeração de fiéis, música e dança, uma festa dentro da festa principal. Em tempo de epidemia, maior era o risco de contágio. Entretanto, o programa de 1919 prometia muita diversão da quinta-feira ao domingo: lavagem, iluminação, quermesse e música até as 22 horas do sábado. No domingo, haveria missas, procissão, e, à noite, quermesse e fogos de planta (Confraria..., 23 out. 1919, p.3).

Os anúncios das celebrações religiosas nos jornais demonstram que até outubro os católicos festejaram seus santos com toda pompa e de acordo com o costume. Apenas em novembro as festas públicas não foram realizadas, somente as missas e procissões penitenciais. Não por acaso, os estudiosos apontam que, “em novembro, o estado era de calamidade pública” (Souza, Hochman, 2012, p.10).

Em 29 de outubro, a mesa administrativa da Ordem Terceira de São Francisco mandou publicar uma nota nos jornais convidando “aos seus caríssimos irmãos, revestidos de seus hábitos e aos fiéis devotos em geral” para acompanhar esse “tão sublime ato de penitência ao Milagroso santo [são Roque], advogado contra a peste” (Dultra, 29 out. 1919, p.2). Assim, no dia primeiro de novembro, quando se comemoram todos os santos, os fiéis privilegiaram o santo medieval que salvou muitas pessoas do contágio da peste negra. O momento exigia um especialista.

No dia 4, o articulista do jornal *O Imparcial* (Varíola..., 4 nov. 1919, p.1) informava que, em apenas três dias, morreram cerca de cem variolosos. A partir dessa data, não há mais nos jornais convites para festas religiosas; porém, são muitos os apelos para a realização de procissões de penitência com as imagens do Senhor do Bonfim e dos tradicionais santos protetores contra as pestes, são Roque e são Lázaro.

Em 7 de novembro, com apenas seis dias de intervalo, foi realizada a segunda procissão de penitência, dessa vez com a imagem do Senhor do Bonfim que pertencia à Ordem Terceira do Carmo. Os fiéis, entretanto, não consideraram o itinerário suficiente, e “inúmeras famílias residentes no distrito de S. Pedro” pediam aos irmãos terceiros do Carmo que realizassem outra procissão, a fim de que a “milagrosa imagem” percorresse “as ruas de toda aquela freguesia que não foram visitadas por ocasião da procissão de ontem” (Senhor..., 8 nov. 1919, p.2).

Diante das dificuldades da Saúde Pública para controlar a epidemia, os clamores a Deus e aos santos em duas procissões penitenciais não se mostraram suficientes para diminuir a dor, a angústia e o medo que a varíola causava na população. Uma matéria do *Diário de Notícias* do dia 11 revelava o medo no título: “A asa negra da morte paira por sobre toda a cidade”. Segundo a reportagem, com informações do Desinfetório Público e dos cemitérios, “em cálculo otimista”, entre 15 de setembro e 10 de novembro, morreram 1.243 soteropolitanos com a doença (A asa..., 7 nov. 1919, p.1).

O articulista do *Diário de Notícias* descreveu a cena noturna do Cemitério Quinta dos Lázaros. Segundo ele, cabia a vinte homens “a tarefa macabra” de abrir as covas rasas durante todo o dia, “sem descanso, sem folga”. “Na porta do cemitério ouvem-se ruídos de vozes e do chicote estalando no ar” durante a chegada de mais carros fúnebres, “carruagens fantasmas”, que “continuam pela noite adentro a sua tarefa tristíssima, fazendo ressoar ingubremente (*sic*) nas pedras da ladeira da Quinta, as suas rodas, quebrando o silêncio sepulcral daquelas paragens sossegadas” (A asa..., 7 nov. 1919, p.1).

Esse relato traz a angústia dos ritos fúnebres abreviados durante uma epidemia de doença transmissível. Não eram permitidos os longos velórios, muitas vezes noturnos, com o caixão aberto, as orações, os cânticos e as velas acesas abrindo caminho e iluminando a passagem espiritual da alma. Na primeira metade do século XX, os mortos ainda eram velados em casa ou nas capelas das associações leigas. Ser membro de uma irmandade ou ordem terceira garantia ao associado o sepultamento cristão no cemitério ou carneiro da associação, mas, principalmente, a execução dos ritos considerados necessários à salvação da alma. Os compromissos reelaborados no período republicano mantêm as obrigações para com os irmãos defuntos: acompanhamento à sepultura com os irmãos usando suas capas e insígnias e missas em sufrágio à alma no sétimo e no trigésimo dia do falecimento (Compromisso..., 1914, p.30-33). Em tempo de epidemia, o sepultamento rápido, sem ritos fúnebres e acompanhamentos dos familiares, amigos e irmãos de fé, causava angústia e mal-estar, sensação do último adeus não dado, do luto não vivido.

Em 12 de novembro, a Ordem Terceira do Carmo avisava que atenderia ao pedido dos fiéis. A procissão, com a imagem do Senhor do Bonfim, seria realizada no dia 15 (Venerável..., 12 nov. 1919, p.5). Um dia antes da procissão de penitência, outra imagem do Cristo crucificado, pertencente à Irmandade do Senhor Bom Jesus dos Passos e Vera Cruz, estaria exposta na igreja da Ordem Terceira de São Domingos, “em adoração do povo a fim de obter da Clemência Divina, a extinção do horrível flagelo da varíola que está assustadoramente dizimando a população” (Penitência, 14 nov. 1919, p.7). Na mesma página há a informação sobre um surto de peste bubônica no Rio de Janeiro e a suspensão de aulas em duas escolas. Segundo o jornal, havia casos da doença no Ceará, em Santos, Vitória e Pelotas, além do litoral argentino. Ou seja, havia motivo para preocupação e reforço do clamor à Providência. E a igreja do Bonfim recebia fiéis, em “romarias e penitências” diariamente, como informa *O Imparcial* (A epidemia..., 29 nov. 1919, p.1).

No final de novembro, entretanto, há mudança no tom das notícias. Não há mais expressões e representações de pavor, mas, sim, de júbilo pelo livramento da morte e graças pela vida, perceptíveis nos convites para missas solenes e festas aos santos. Assim, a Irmandade de Santa Cecília convidava os fiéis para a missa solene da festa da padroeira dos músicos, que seria realizada na igreja da Sé no dia 22 (Irmandade..., 20 nov. 1919, p.7). Em meio ao clima de graça alcançada, a varíola continuava fazendo vítimas. A mesma página que anunciava a festa denunciava “a miséria das ruas” (A miséria..., 20 nov. 1919, p.7), ao relatar que naquele dia, na praça 15 de Novembro, “um varioloso moribundo, soltando aos pedaços, exalava o derradeiro alento”, abandonado “na mais absoluta indiferença”. Para finalizar, a pergunta: “E o que nos dirá a Saúde Pública das condições em que ficou aquele banco infeccionado?” (p.7).

Ainda para render graças, em 27 de novembro, uma comissão da igreja de santo Antonio da Barra convidava todos os fiéis para uma missa, no dia 30, “em ação de graças ao mesmo santo por ter sido aquele arrabalde, o mais poupado pela peste que flagela. É esta uma boa ocasião dos corações gratos manifestarem o seu reconhecimento ao nosso glorioso Taumaturgo” (Em Santo..., 27 nov. 1919, p.7). Também em agradecimento pelo fim da epidemia, a mesma comissão preparava uma missa de ação de graças a são Lázaro, em sua igreja, no dia 14 de dezembro.

A alegria das festas religiosas voltou em dezembro. No primeiro dia do mês, a Irmandade do Santíssimo Sacramento e Nossa Senhora da Conceição da Praia já anunciava a programação da festa da padroeira do Brasil em coluna inteira do *Diário de Notícias*. Missas seriam celebradas de hora em hora, com orquestra e coral. A procissão juntaria os membros da irmandade, além dos irmãos da Irmandade do Senhor Bom Jesus da Redenção e da Irmandade de Nossa Senhora do Rosário. O préstito percorreria um longo percurso, do distrito comercial, com passagem pelo terreiro de Jesus até a praça da Piedade, de onde retornaria ao templo (Queiroz, 1 dez. 1919, p.3). A festa de santa Luzia foi celebrada “com toda a solenidade e pompa, de costume”, na igreja do Pilar (Festa..., 12 dez. 1919, p.2).

Começaram, em seguida, os festejos da natividade de Jesus. A Ordem Terceira do Carmo, que tanto se dedicou aos atos penitenciais durante a epidemia, anunciava a festa de largo, a missa do galo e convidava os fiéis à adoração do Menino Jesus no presépio armado dentro da igreja (Venerável..., 23 dez. 1919, p.4). Assim, nas celebrações do fim do ano, os fiéis passaram do sofrimento simbolizado pela invocação do Senhor do Bonfim à alegria do nascimento de Jesus, à renovação da vida. Dessa forma, o Natal foi celebrado com júbilo e esperança de dias melhores em 1920.

## Considerações finais

O Senhor do Bonfim foi a quem mais os católicos soteropolitanos recorreram, em ritos religiosos públicos e coletivos, na busca da interseção para aplacar a dor e salvar vidas durante as epidemias de gripe espanhola, varíola e covid-19. Desde o período colonial, o culto ao Cristo crucificado se configurou como objeto da maior devoção em Salvador. Em 1919, das oito associações leigas criadas para esse fim no Setecentos, apenas uma, a Irmandade do Senhor Bom Jesus dos Passos dos Humildes, tinha sido extinta, em 1909. A Ordem Terceira do Carmo possuía também uma imagem do Crucificado considerada milagrosa. Foram realizadas quatro procissões penitenciais – uma para são Roque, outra para são Lázaro e duas para o Senhor do Bonfim –, sendo que uma imagem ficou exposta à visitação na igreja da Ordem Terceira de São Domingos.

A análise da documentação sobre as atividades religiosas desenvolvidas ou suspensas durante as epidemias de gripe espanhola e varíola demonstrou diferentes reações dos católicos vinculados às associações leigas. Em 1918, as festas públicas aconteceram “com a pompa habitual”, enquanto em 1919 foram suspensas ou substituídas por missas e procissões de penitência.

A demora das autoridades civis e de saúde pública para decretar que aquela não era uma gripe comum, tomar medidas profiláticas e instruir a população para fazer o isolamento social dava a sensação de normalidade e segurança, inclusive para a reunião de dezenas ou centenas de devotos em espaços fechados, como as igrejas. Além disso, a tuberculose assustava muito mais, com alto índice de mortalidade. Em 1918, enquanto a gripe espanhola matou 386 pessoas, a tuberculose fez 1.153 vítimas. Portanto, era difícil convencer a população de que uma doença, considerada benigna, necessitasse de isolamento. Pelo contrário, para os religiosos, os ritos coletivos seriam eficazes para a cura. Assim, as festas não foram consideradas aglomerações favoráveis à disseminação da doença.

Já a varíola era uma doença bem conhecida da população baiana, com surtos em cada década. Apesar da existência da vacina, muitos rejeitavam essa medida; porém, sabiam do alto grau de mortalidade, sendo 2.804 óbitos em 1919. Além disso, a presença de um varioloso, com suas feridas expostas, causava repugnância e pavor. Era preciso, então, precaver-se contra o mal. Os articulistas dos jornais de Salvador denunciavam o descaso do governo estadual com a saúde e a situação de miséria da população e da municipalidade com o asseio das ruas, clamavam por medidas de isolamento dos doentes e representavam, por meio do discurso e de imagens em tons dramáticos, o medo da morte. Nessa epidemia, o isolamento foi mais observado; os festejos públicos, suspensos; e os ritos penitenciais, realizados para clamar pela Providência divina.

## Data Availability

Os dados da pesquisa foram coletados nos arquivos públicos referendados no artigo e não disponibilizados em arquivos digitais.

## NOTAS

1 O compromisso da Confraria do Senhor Bom Jesus da Cruz, reelaborado em 1874 e reimpresso em 1914, traz, em seu capítulo I, um histórico da fundação da confraria, ocorrida em 1719, quando caía uma forte tempestade sobre a cidade do Salvador. “Um homem, de cor parda, em hábitos de santa penitência, apresenta-se nas ruas desta cidade abraçado com a Santa Cruz” e convoca os moradores para uma procissão. O relato desse fato determina o nome da associação leiga e o critério étnico de admissão dos seus membros: homens pardos (Compromisso..., 1914, p.5).
